# Familial Aggregation of Primary Hyperparathyroidism and Malignancy—Nationwide Case-Control and Cohort Study

**DOI:** 10.1210/jendso/bvaf152

**Published:** 2025-09-23

**Authors:** David Thorsteinsson, Fredrik Granath, Robert Bränström, Jan Zedenius, Inga-Lena Nilsson

**Affiliations:** Department of Breast, Endocrine Tumors and Sarcoma, Karolinska University Hospital, 171 76 Stockholm, Sweden; Department of Molecular Medicine and Surgery, Karolinska Institutet, 171 76 Stockholm, Sweden; Department of Medicine Solna, Division of Clinical Epidemiology, Karolinska Institutet, 171 76 Stockholm, Sweden; Department of Breast, Endocrine Tumors and Sarcoma, Karolinska University Hospital, 171 76 Stockholm, Sweden; Department of Molecular Medicine and Surgery, Karolinska Institutet, 171 76 Stockholm, Sweden; Department of Breast, Endocrine Tumors and Sarcoma, Karolinska University Hospital, 171 76 Stockholm, Sweden; Department of Molecular Medicine and Surgery, Karolinska Institutet, 171 76 Stockholm, Sweden; Department of Breast, Endocrine Tumors and Sarcoma, Karolinska University Hospital, 171 76 Stockholm, Sweden; Department of Molecular Medicine and Surgery, Karolinska Institutet, 171 76 Stockholm, Sweden

**Keywords:** hyperparathyroidism, malignancy, familial, genetics, case-control, cohort

## Abstract

**Context:**

Primary hyperparathyroidism (PHPT) presents both in sporadic and hereditary forms, with familial clustering observed in certain genetic syndromes. While emerging research suggests an increased malignancy risk in patients, the extent to which this association extends to their first-degree relatives remains unclear. Understanding familial aggregation of PHPT and malignancies could reveal underlying genetic risk factors and guide clinical management.

**Objective:**

This work aimed to assess familial clustering of PHPT and malignancies among first-degree relatives of affected patients.

**Methods:**

A nationwide register-based case-control and cohort study was conducted including all patients with PHPT who underwent parathyroidectomy between 2008 and 2017, with matched controls and their first-degree relatives. This Swedish, nationwide, population-based register study included 6693 patients born in Sweden after 1932 who were matched with 33 393 controls. Main outcome measures included diagnoses of PHPT and malignancies among first-degree relatives.

**Results:**

A total of 218 729 first-degree relatives were identified. Relatives of patients had statistically significantly higher odds of PHPT, particularly if diagnosed at age 45 years or younger (odds ratio [OR] 7.7; 95% CI, 5.23-11.34; *P* < .001). The risk of malignancy was slightly increased (OR 1.07; 95% CI, 1.01-1.13; *P* = .017), due to prostate, nonmedullary thyroid, and hematologic malignancies. In prospective analysis, no increased risk of malignancy in relatives was observed.

**Conclusion:**

This study highlights a significant familial aggregation of PHPT, particularly in early-onset cases. Although a modest overrepresentation of a family history of malignancy was observed, this may reflect multiple comparisons and surveillance bias rather than a true causal link between PHPT and cancer.

The annual incidence of parathyroid tumors has been estimated as 4 to 6 patients per 10 000 person-years [1]. The majority of these occur sporadically but familial forms exist and have been estimated to account for between 5% and 15% of all cases of primary hyperparathyroidism (PHPT) [2, 3]. Familial forms of PHPT occur isolated (FIHPT) or as part of a syndrome [4]. The distinction between FIHPT and syndromic forms is often unclear and depends on the extent of family history available and the possible occurrence of de novo germline mutations. Some syndromes involve well-established autosomal dominant genetic pathways linking parathyroid adenoma development with neoplasia [3, 5, 6]. The most common syndromic form of PHPT is multiple endocrine neoplasia type 1 (MEN1) in which the penetrance of PHPT is approximately 90%. Other hereditary autosomal dominant syndromes include MEN2A, familial hypocalciuric hypercalcemia, neonatal-severe hyperparathyroidism, and hyperparathyroidism-tumor-jaw syndromes. In FIHPT, pathogenic genetic variants commonly associated with syndromic forms of PHPT, such as *MEN1* and *CDC73*, are identified in approximately one-third, suggesting partial manifestation of syndromic PHPT [7]. Germline mutations in *GCM*2, a regulator of parathyroid development, have been observed in FIHPT but also in sporadic forms of PHPT [8]. Other mutations have been reported and familial forms of PHPT remain to be explored and characterized [9]. Also, since sporadic PHPT is relatively common, clusters of PHPT diagnoses within small families may result from coincidence rather than hereditary factors [7].

Earlier studies have demonstrated an association between parathyroid adenoma and malignancy beyond the established syndromic pathways and even for mild PHPT [10-18]. A recent meta-analysis reported a pooled prevalence of 20% for any type of cancer among individuals with PHPT, with papillary thyroid cancer and breast cancer being the most common [19]. Other cancers found to be overrepresented in patients with PHPT included gastric cancer, basal cell carcinoma, prostate cancer, colorectal cancer, urinary tract cancer, and hematologic malignancies. The overall relative risk increase of developing malignancies was about 28% compared to controls [19]. This suggests the possibility of previously unidentified somatic mutations in patients with PHPT that may be linked to cancer development like known syndromic PHPT genotypes.

The authors hypothesize that there exists an increased familial aggregation of PHPT (primary end point) and malignancy (secondary end point) in first-degree relatives of patients with PHPT outside the known syndromic pathways.

## Materials and Methods

This report adheres to the Strobe guidelines for observational studies [20]. It was designed as a population-based, nationwide investigation comprising two components: a retrospective case-control study and a prospective cohort component. The characteristics of patients and controls have been described in detail previously [21]. In brief, all patients in Sweden who underwent parathyroidectomy for PHPT between January 1, 2008, and December 31, 2017, were identified through the Scandinavian Quality Register for Thyroid, Parathyroid, and Adrenal Surgery (SQRTPA) and the Swedish National Cancer Register (SCR). Patients with secondary or tertiary hyperparathyroidism are not registered in the SCR and were not included from the SQRTPA dataset. In the original dataset, population controls were selected and matched by Statistics Sweden (SCB) according to year of parathyroidectomy, age, sex, and address, with a 10:1 ratio of controls to each patient. The dataset was further expanded by linking it to the Swedish Multi-Generation Register (MGR) using personal identity numbers. The MGR is part of the Total Population Register, drawing information from the National Tax Board and updated annually. Its primary purpose is to support epidemiological research, and it is maintained by SCB, which enables linkage with other population registers via personal identity numbers. The MGR contains data on individuals born in Sweden in 1932 or later, as well as those registered in Sweden at any time since 1961, along with their biological or adoptive parents [22, 23]. A first-degree relative was defined as a biological mother, father, full sibling (sharing both parents), or child. Each case and control (index person) was linked to all first-degree relatives in the dataset, with a maximum of 25 relatives per individual. Children were defined as offspring born before the index date of parathyroidectomy. The type, sex, and birth year of each relative were recorded. By linking to the SCR and the National Patient Register, information was obtained on relatives' dates of diagnosis and diagnoses of malignant neoplasms. A comprehensive list of included diagnostic codes is provided in Supplementary Tables S1 and S2 [24]. In the case-control component of the investigation, a family history of PHPT or malignancy was treated as the exposure, and parathyroidectomy for PHPT as the outcome. To enable the investigation of events occurring after the date of parathyroidectomy, the cohort of patients with PHPT who underwent parathyroidectomy (exposure) was followed prospectively over time, with the subsequent occurrence of PHPT recorded as the primary outcome. The primary end point of both components was the diagnosis of PHPT in a first-degree relative of the patients and their respective controls. Systematized Nomenclature of Medicine (SNOMED) codes were used to exclude medullary thyroid neoplasms from the thyroid neoplasm category. The remaining cases and category are hereafter referred to as nonmedullary thyroid cancers (NMTCs). SNOMED codes also allowed for the identification and exclusion of pancreatic islet cell neoplasm from the pancreas neoplasm category. These were instead classified under endocrine organ neoplasia, which includes both malignant and benign neoplasms of pituitary, parathyroid, pancreatic islet cells, and adrenal glands. Assumed syndromic familial forms of PHPT were then defined as the coexistence of PHPT and endocrine organ neoplasia. The secondary end points of both components of the study were diagnoses of malignant neoplasms by anatomical site and endocrine neoplasia in first-degree relatives. Information on germline genetic testing was not available.

In this study, patients born outside Sweden and patients born before 1932 were excluded from the dataset as these individuals were not captured by the MGR. Thus, only patients and controls with a complete family history were included. This exclusion created a mismatch of available controls for patients based on country of birth (patients and controls were not matched on this variable). To offset this imbalance in number of controls, cases with more than 5 remaining controls after exclusion were randomly assigned 5 controls from the respective control group in the same matched set. Cases with 5 controls or fewer retained all available matched controls. This resulted in 99.2% of the cases having 5 controls and only 0.8% with between 2 and 4 controls (flow diagram 1 shows the number of patients examined for eligibility and the distribution of matched controls). To enable comparisons of diagnoses over time, more recent International Classification of Diseases, Tenth Revision (ICD-10) codes have been back-coded to ICD-7 by SCR. The overall completeness of SCR is high [25]. Each individual in the dataset, including index individuals and their first-degree relatives, was assigned a unique serial number and pseudonymized by SCB before being provided to researchers. Patients and controls were organized into matched sets using set numbers.

Summary statistics were used to present counts and percentages, medians with interquartile range, or means with SD, as appropriate. Frequency differences in contingency tables were analyzed using the Pearson chi-square test. In the case-control component of the study, a conditional logistic regression model was applied to matched sets of patients and controls, with case or control status as the dependent variable and tumor diagnoses as independent variables. Subset analyses for age and sex were performed and reported. For the prospective cohort component of this study, a Cox proportional hazards model was used to estimate the incidence of PHPT, endocrine organ neoplasia, and malignancies in all anatomical sites, in first degree relatives, with stratification by birth-year, sex, and year at start of follow-up. Individuals were censored at the time of death or at end of follow-up, whichever occurred first. Results of regression models are presented as odds ratios (ORs) or hazard ratios (HRs), respectively, with corresponding 95% CIs, using a 2-tailed α level of .05. Assuming the rare disease model, OR and risk are used interchangeably throughout the text. Statistical analyses, graphs, and maps were generated using RStudio (version 2021.09.2 + 382) with the Tidyverse package (version 1.3.1) [26]; logistic regression analysis was conducted using the Survival Package [27]. Forest plots were created with the Metafor package [28]. Ethical approval was obtained from the Swedish Ethical Review Authority prior to data collection (Dnr. 2019-02149).

## Results

**Figure 1.**
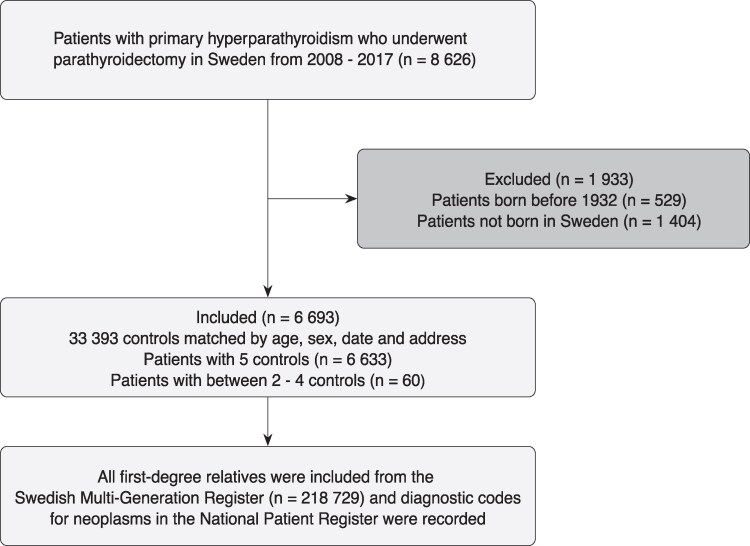
Flow diagram outlining study participant inclusion, eligibility, number of matched controls, and first-degree relatives.

The total number of patients and controls with complete family history was 6693 and 33 393, respectively. Among these, 36 386 first-degree relatives of patients and 182 343 first-degree relatives of controls were identified and included in the analysis. Fig. 1 outlines the patient selection process, criteria, and counts for inclusion and exclusion. Supplementary Table S3 [24] presents the demographics and distribution of first-degree relatives of patients and controls, demonstrating virtually identical family structures between the groups.

**Figure 2.**
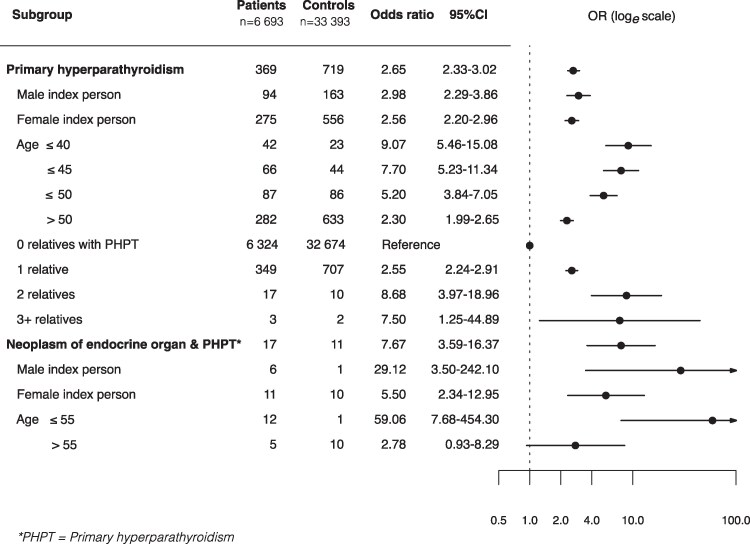
Forest plot illustrating odds ratios for primary hyperparathyroidism and endocrine organ neoplasms among first-degree relatives of patients with primary hyperparathyroidism compared with controls.

**Figure 3.**
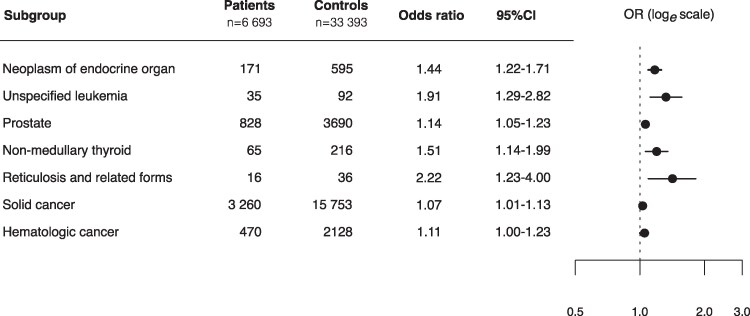
Forest plot showing odds ratios for malignancy and endocrine organ neoplasms among first-degree relatives of patients with primary hyperparathyroidism compared with controls.

The prevalence of PHPT family history among cases was almost 3 times higher among patients operated on for PHPT compared to their matched controls, and this association was about the same for male and female index patients (Fig. 2). Additionally, Fig. 2 also shows that family history for PHPT is increasingly more common with the decreasing age of the patients and that the association strengthens with an increasing number of relatives with PHPT. Family history defined as having at least 1 family member diagnosed both with PHPT and a neuroendocrine neoplasia is about 8 times more common among patients than among controls, while the odds increase for only having a relative with a neuroendocrine tumor is about 40%. These associations are illustrated in Fig. 3, which presents the OR for the association between family history of malignant neoplasms, endocrine organ neoplasia, and PHPT.

**Figure 4.**
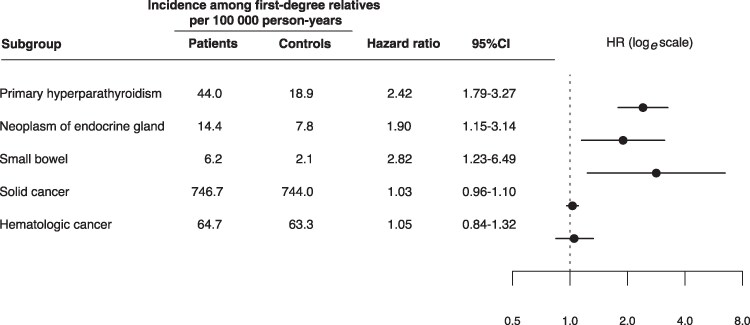
Forest plot illustrating hazard ratios for primary hyperparathyroidism, endocrine organ neoplasms, and malignancies among first-degree relatives of patients compared with controls.

Patients with PHPT had slightly higher odds of having a first-degree relative with a malignancy compared with controls (OR 1.07; 95% CI, 1.01-1.13). Prostate cancer, NMTC, and hematologic malignancies were slightly more common in the families of patients with PHPT undergoing parathyroidectomy. Supplementary Table S1 [24] provides OR, 95% CI, and *P* values for the family history of all individual tumor sites as estimated from the case-control conditional regression models. As shown in Fig. 4, Cox proportional hazards models demonstrated an increased hazard for PHPT (HR 2.42; 95% CI, 1.79-3.27) and endocrine organ neoplasia (HR 1.90; 95% CI, 1.15-3.14) in first-degree relatives of patients with PHPT. The HR for all solid cancers and hematologic malignancies was 1.03 (95% CI, 0.96-1.10) and 1.05 (95% CI, 0.84-1.32), respectively. A statistically increased hazard of small bowel malignancy was also observed in first-degree relatives of patients (HR 2.82; 95% CI, 1.23-6.49), whereas no significant difference in hazard was found for malignancies at other anatomical sites.

However, no associations with malignancy in either model remained statistically significant after correction for multiple testing using a false discovery rate of .05. Detailed results from the Cox proportional hazards and conditional logistic regression analyses are provided in Supplementary Table S2 [24]

## Discussion

### Key Results

This study is the first large-scale investigation of familial aggregations of PHPT and its association with malignant neoplasms. The findings confirm a notable familial aggregation of PHPT, particularly among patients younger than 45 years. In contrast, the observed familial aggregation of malignancies was lower than anticipated. Current European expert consensus guidelines recommend genetic counseling for patients with PHPT who are younger than 30 years [29]. In contrast, Swedish guidelines and the American Association of Endocrine Surgeons' guidelines set the age threshold for genetic counseling at 40 years [30, 31]. The results of this study support an age threshold of 40 years for genetic counseling, which could enhance the identification of familial PHPT.

The index patients with PHPT had a significantly increased risk of having relatives with combinations of PHPT and neuroendocrine tumors. The genetic pathways associated with these neoplasms are well documented [2, 5, 32]. This risk increase was highest for male patients at any age (OR 29.12; 95% CI, 3.50-242.10) and all patients aged 55 or younger with an OR of 59.06 (95% CI, 7.68-454.30), aligning with the natural history of MEN1, the predominant syndromic form of familial PHPT, which typically presents in young adulthood. Relatives with a combined concurrent diagnosis of PHPT and neuroendocrine tumors were not included in the regression analysis of PHPT counts between patients and control groups, and including these patients would further inflate the odds difference of having a relative with PHPT between patients and controls. Thus, the substantial difference in OR for PHPT diagnoses between patients and controls cannot be attributed to the known syndromic pathways. Also, it is widely accepted that the majority of PHPT cases occur as nonfamilial solitary endocrinopathies, and the prevalence of FIHPT is probably underestimated [5]. These patients exhibit an autosomal dominant hereditary form of PHPT without evidence of other endocrinopathies. Distinguishing between these two clinical entities is often challenging, as it depends on a thorough family history and on family members living long enough to manifest and present with symptoms. It is also possible that FIHPT may be explained by de novo germline mutations, which could subsequently be passed on to the offspring of these patients, thereby explaining the lack of family history in the proband [2].

### Malignancies

The case-control analysis of this study showed a modest overrepresentation of malignancies among first-degree relatives of patients with PHPT selected for parathyroidectomy. A recent meta-analysis by Charoenngam et al [19] reported a substantially higher pooled prevalence of malignant neoplasms in patients with PHPT and highlighted the scarcity of research on potential hereditary links between PHPT and malignancy [19]. In the present study, the prospective cohort analysis demonstrated a minor increase in hazard of small bowel malignancies among first-degree relatives of patients with PHPT; however, this association did not persist after correction for multiple testing. No difference in hazard was observed for malignancies at other anatomical sites. These findings suggest that hereditary factors beyond the known genetic pathways of syndromic PHPT are likely to play only a limited role in this association.

Prostate cancer was the most common malignant neoplasm in first-degree relatives in this study and was overrepresented in family members of patients selected for parathyroidectomy (OR 1.14; 95% CI, 1.05-1.23). Minisola and colleagues [14] recently demonstrated a high prevalence of PHPT in men with prostate cancer. The etiology of this association remains unclear. Environmental and hereditary pathways between PHPT and prostate cancer have been suggested. One proposed mechanism has been the increased production of fibroblast growth factor 23 by prostate cells in reaction to hypophosphatemia caused by PHPT [33]. However, the effect of surveillance bias is probably an important confounder.

A significantly elevated risk of NMTC among relatives of patients with PHPT (OR 1.51; 95% CI, 1.14-1.99) was observed. While surveillance bias often complicates results, numerous studies have reported an increased risk of concurrent NMTC and PHPT, whereas others have failed to confirm this association [3, 10, 13, 34-36]. Increased diagnostic intensity is anticipated in patients with PHPT given the extensive neck imaging routinely performed as part of preoperative evaluation in those undergoing parathyroidectomy. Consequently, a detection bias may arise, potentially inflating the observed OR. However, the effect of detection bias does not fully account for the relatively large OR of NMTC diagnoses among first-degree relatives. This finding lends support to the hypothesis that genetic and environmental factors may contribute, albeit weakly, to a shared predisposition of PHPT and NMTC.

Nilsson and colleagues [10] have reported a high standardized incidence ratio of breast cancer diagnoses among patients with PHPT selected for parathyroidectomy. This finding is corroborated by a systematic review by Charoenngam et al [19] showing a higher-than-expected pooled prevalence of 5% for breast cancer in patients with PHPT. This study found no difference in the prevalence of breast cancer in first-degree relatives of patients compared with controls, suggesting any genetic pathway between these two entities is unlikely. The persistent risk increase over time after curative parathyroid surgery suggests any causative link to direct or indirect effects of hypercalcemia is also unlikely [12 , 37].

Hematologic malignancies among first-degree relatives were marginally overrepresented in the patient cohort (OR 1.11; 95% CI, 1.00-1.23), primarily due to a higher prevalence of essential thrombocythemia diagnoses among relatives of patients. The diagnosis of other and unspecified leukemia was found in almost twice as many first-degree relatives of patients compared with controls (OR 1.91; 95% CI, 1.29-2.82). Previous research has indicated an increased risk of hematologic malignancies among patients with PHPT, with Pickard et al [13] reporting a standardized incidence ratio of 1.88 (95% CI, 1.0-3.2) based on 13 patients where the excess risk was driven primarily by 4 patients with multiple myeloma. No overrepresentation of multiple myeloma among first-degree relatives of patients with PHPT was observed in this study.

Finally, neither the case-control nor the cohort components of the study identified differences in OR or HR for pancreatic, colorectal, gastric, or gynecological malignancies in first-degree relatives of patients and controls, suggesting that a shared genetic link between PHPT and these cancers is unlikely.

### Strengths and Limitations

This study's strengths include the large number of first-degree relatives captured within the MGR, which provides robust statistical power, and the comprehensive accuracy of both the MGR and the SCR with nationwide coverage from 1932 and 1958, respectively. An additional strength is this study’s bidirectional design, which allows for the assessment of familial clustering both retrospectively and prospectively from the index date. However, the study has certain limitations. The use of a case-control design in which patients undergoing parathyroidectomy were used as proxies for the general population of individuals with PHPT may introduce detection bias and limit external validity. Moreover, complete National Patient Register coverage of tertiary care visits was not achieved until 2001, making underreporting of PHPT among relatives likely. Such underreporting would introduce nondifferential misclassification, leading to underestimation of familial PHPT both in patients and controls. By contrast, underreporting is expected to be minimal for cancer diagnoses, as the SCR has provided high coverage and accurate coding since its inception in 1958 [25].

The diagnosis of PHPT is reliant on laboratory testing and is inherently linked to health-seeking behavior, which may itself be a familial trait. This mechanism could lead to an earlier detection of malignancies among relatives of patient with PHPT, resulting in an overrepresentation of diagnosed malignancies in this group. This potential bias may be particularly relevant for malignancies detected through diagnostic tests rather than symptoms, such as prostate cancer and essential thrombocythemia. Diagnosis of a family member could also increase health-seeking behaviors among patients, which in turn may enhance the likelihood of detecting and treating preexisting PHPT, although this is likely to have a lesser effect on their first-degree relatives. Based on prior analyses of this dataset, the authors hypothesize that the observed overrepresentation of neoplasms among first-degree relatives may, in part, reflect differences in health-seeking behavior. This pattern has been particularly apparent in comparisons between patients who underwent parathyroidectomy and their matched controls [21]. However, as data on health-care utilization among first-degree relatives are not available, this hypothesis cannot be directly evaluated. Health-seeking behavior is therefore proposed as a potential explanatory factor, while acknowledging that this interpretation remains speculative given the limitations of the available data.

Also, multiple separate and simple regression models were performed simultaneously to assess the association between ICD-7 cancer diagnoses in relatives and case status as the outcome variable. While this method enables the identification of potential associations, it is critical to acknowledge that conducting multiple family-wise statistical comparisons simultaneously increases the probability of false-positive results, and after applying a false discovery rate of .05 on the individual site-specific *P* values, no association remains statistically significant. Moreover, exposures to rare diagnoses may yield unstable estimates with wide CIs, further contributing to statistical artifacts. Therefore, caution is warranted in interpreting individual ORs, and results should be contextualized within the broader biological and epidemiological framework.

This study reported an aggregation of PHPT diagnoses in family members of patients selected for surgical treatment of PHPT, suggesting a true familial risk. Earlier onset was associated with higher odds of familial PHPT, which could partly reflect genetic anticipation, the phenomenon in which a genetic disorder manifests at an earlier age or with increased severity in successive generations. Anticipation is most clearly demonstrated in neuropsychiatric disease, for example, in trinucleotide repeat disorders and telomere biology disorders, but has also been reported in hereditary cancer syndromes [38, 39]. Evidence of anticipation has been described in MEN1 families [40], although its existence and relevance in nonsyndromic familial PHPT remain largely unexplored. Retrospective analyses of anticipation are vulnerable to bias, since younger generations are often diagnosed earlier through increased surveillance or during evaluation of their affected parents, while older generations are more prone to truncation and misclassification bias due to incomplete health registers. These factors can create an artificial impression of earlier onset in later generations. The potential contribution of anticipation to familial PHPT warrants further investigation, ideally through studies that combine register data with germline genetic testing.

### Generalizability

The demographic profile of the patients in this study is comparable to that reported in a large Scottish population-based incidence study of PHPT during a similar period [1]. Moreover, as previously reported by this research group, approximately 74% of operated-on patients met national guideline criteria (serum calcium ≥1.45 mmol/L, nephrolithiasis, osteoporosis, or age <50 years), while 16% of those who underwent parathyroidectomy lacked such indications and thus had relatively mild disease [21]. The cohort's median serum calcium of 1.44 mmol/L (range, 1.14-2.62 mmol/L) further indicates that it was not restricted to the most biochemically severe cases but included a broad spectrum of disease severity. Still, we cannot exclude the possibility that unoperated-on patients would show an association with the occurrence of malignancy among first-degree relatives. However, given the broad range of disease severity in this cohort and the substantial proportion of patients with mild PHPT, this potential phenotype is at least partly represented in the data.

Furthermore, defining the true source population for PHPT is inherently difficult, as the disorder spans a continuum of biochemical abnormalities in calcium and PTH homeostasis. Patients fulfilling surgical criteria but not operated on are often older or frailer, and excluding them is effectively conditional on age and comorbidity, both factors that may themselves influence malignancy risk. Likewise, not sampling patients with very mild disease who do not meet surgical indications could introduce ascertainment bias.

Consequently, restricting analyses to surgically treated PHPT could bias estimates upward by overrepresenting patients with greater disease burden who are fit for surgery. Importantly, however, the results did not reveal strong associations but only modestly increased risk of malignancy, which argues against substantial inflation due to surgical selection. We therefore conclude that while our findings are most directly generalizable to surgically treated PHPT, they remain informative for understanding the relationship between PHPT and malignancy in the source population.

### Interpretation

Patients with PHPT selected for parathyroidectomy have a higher frequency and OR of having relatives with PHPT. A total of 6% of patients with PHPT had 1 or more first-degree relatives with PHPT without a known syndromic form compared with 2% of controls. The cutoff age for genetic testing and counseling should be set at a minimum of 40 years, as the risk of having a first-degree relative affected is almost 10 times higher in this age group.

This study does not rule out the hypothesis of hitherto unidentified germline mutations acting as a causative link between parathyroid adenoma and some malignant tumors, but this association, if it exists, can be inferred to be weak. Malignancies that are overrepresented in patients with PHPT (colon cancer, gastric cancer breast cancer, multiple myeloma, urinary tract malignancies, and skin cancer) were not more common in first-degree relatives of patients with PHPT operated on with parathyroidectomy [10, 11, 13, 16]. Given the high statistical power and the robust quality of data in this study, the associations observed earlier between PHPT and these malignancies in patients are unlikely to be attributable to inherited germline mutations contributing both to PHPT and tumor development.

This study suggests a weak familial association between PHPT and certain malignancies. While slightly elevated ORs were observed for malignancies such as prostate cancer, NMTC, and hematologic malignancies in first-degree relatives of patients with PHPT, the overall risk of malignancy remains limited and is likely influenced by multiple factors. These include the effects of performing multiple simultaneous simple regression models as well as potential surveillance bias in relatives of patients undergoing parathyroidectomy. Furthermore, prospective follow-up revealed only a marginally increased hazard of having a relative diagnosed with small bowel cancer, a finding that did not remain statistically significant after adjusting for multiple comparisons. Taken together, these results do not provide evidence of a significantly increased risk of malignancy in first-degree relatives of patients with PHPT who underwent parathyroidectomy.

In conclusion, these findings highlight an increased risk of familial nonsyndromic PHPT in younger patients with PHPT undergoing parathyroidectomy. The results underscore the importance of family history, genetic screening, and counseling in selected patients with PHPT, particularly those diagnosed at or before age 40 years, to identify a potential hereditary form of the disease. Future research employing large-scale genetic sequencing could further elucidate these associations, address confounding factors, and support more individualized approaches to clinical assessment, surveillance, and management of this common endocrinopathy.

## Data Availability

Restrictions apply to the availability of some, or all data generated or analyzed during this study to preserve patient confidentiality or because they were used under license. The corresponding author will on request detail the restrictions and any conditions under which access to some data may be provided.

## Abbreviations

FIHPT: familial isolated primary hyperparathyroidism
HR: hazard ratio
ICD: International Classification of Diseases
MEN1: multiple endocrine neoplasia type 1
MGR: Swedish Multi-Generation Register
NMTC: nonmedullary thyroid cancer
OR: odds ratio
PHPT: primary hyperparathyroidism
PTH: parathyroid hormone
SCB: Statistics Sweden
SCR: Swedish National Cancer Register
SNOMED: Systematized Nomenclature of Medicine
SQRTPA: Scandinavian Quality Register for Thyroid, Parathyroid, and Adrenal Surgery

## References

[1] Soto-Pedre E, Newey PJ, Leese GP. Stable incidence and increasing prevalence of primary hyperparathyroidism in a population-based study in Scotland. J Clin Endocrinol Metab. 2023;108(10):e1117‐e1124. 37022975 10.1210/clinem/dgad201 PMC10505547

[2] Iacobone M, Carnaille B, Palazzo FF, Vriens M. Hereditary hyperparathyroidism—a consensus report of the European society of endocrine surgeons (ESES). Langenbecks Arch Surg. 2015;400(8):867‐886. 26450137 10.1007/s00423-015-1342-7

[3] Cetani F, Dinoi E, Pierotti L, Pardi E. Familial states of primary hyperparathyroidism: an update. J Endocrinol Invest. 2024;47(9):2157‐2176. 38635114 10.1007/s40618-024-02366-7

[4] DeLellis RA, Mangray S. Heritable forms of primary hyperparathyroidism: a current perspective. Histopathology. 2018;72(1):117‐132. 29239035 10.1111/his.13306

[5] Thakker RV . Genetics of parathyroid tumours. J Intern Med. 2016;280(6):574‐583. 27306766 10.1111/joim.12523

[6] English KA, Lines KE, Thakker RV. Genetics of hereditary forms of primary hyperparathyroidism. Hormones (Athens). 2024;23(1):3‐14. 38038882 10.1007/s42000-023-00508-9 PMC10847196

[7] Blau JE, Simonds WF. Familial hyperparathyroidism. Front Endocrinol (Lausanne). 2021;12:623667. 33716975 10.3389/fendo.2021.623667 PMC7947864

[8] Guan B, Welch JM, Sapp JC, et al GCM2-Activating mutations in familial isolated hyperparathyroidism. Am J Hum Genet. 2016;99(5):1034‐1044. 27745835 10.1016/j.ajhg.2016.08.018 PMC5097944

[9] Nilsson I-L . Primary hyperparathyroidism: should surgery be performed on all patients? Current evidence and residual uncertainties. J Intern Med. 2019;285(2):149‐164. 30289185 10.1111/joim.12840

[10] Nilsson IL, Zedenius J, Yin L, Ekbom A. The association between primary hyperparathyroidism and malignancy: nationwide cohort analysis on cancer incidence after parathyroidectomy. Endocr Relat Cancer. 2007;14(1):135‐140. 17395982 10.1677/erc.1.01261

[11] Feig DS, Gottesman IS. Familial hyperparathyroidism in association with colonic carcinoma. Cancer. 1987;60(3):429‐432. 3594382 10.1002/1097-0142(19870801)60:3<429::aid-cncr2820600325>3.0.co;2-3

[12] Michels KB, Xue F, Brandt L, Ekbom A. Hyperparathyroidism and subsequent incidence of breast cancer. Int J Cancer. 2004;110(3):449‐451. 15095313 10.1002/ijc.20155

[13] Pickard AL, Gridley G, Mellemkjae L, et al Hyperparathyroidism and subsequent cancer risk in Denmark. Cancer. 2002;95(8):1611‐1617. 12365007 10.1002/cncr.10846

[14] Minisola F, Cipriani C, Colangelo L, et al Mineral metabolism abnormalities in patients with prostate cancer: a systematic case controlled study. Endocrine. 2018;59(2):338‐343. 28660378 10.1007/s12020-017-1351-0

[15] Palmer M, Adami HO, Krusemo UB, Ljunghall S. Increased risk of malignant diseases after surgery for primary hyperparathyroidism. A nationwide cohort study. Am J Epidemiol. 1988;127(5):1031‐1040. 3358404 10.1093/oxfordjournals.aje.a114879

[16] Palmieri S, Roggero L, Cairoli E, et al Occurrence of malignant neoplasia in patients with primary hyperparathyroidism. Eur J Intern Med. 2017;43:77‐82. 28595761 10.1016/j.ejim.2017.06.001

[17] Hedbäck G, Odén A. Increased risk of death from primary hyperparathyroidism--an update. Eur J Clin Invest. 1998;28(4):271‐276. 9615902 10.1046/j.1365-2362.1998.00289.x

[18] Yu N, Donnan PT, Flynn RW, et al Increased mortality and morbidity in mild primary hyperparathyroid patients. The parathyroid epidemiology and audit research study (PEARS). Clin Endocrinol (Oxf). 2010;73(1):30‐34. 20039887 10.1111/j.1365-2265.2009.03766.x

[19] Charoenngam N, Rittiphairoj T, Wannaphut C, Pangkanon W, Saowapa S. Risk of malignant neoplasm in patients with primary hyperparathyroidism: a systematic review and meta-analysis. Calcif Tissue Int. 2024;115(1):1‐13. 38772934 10.1007/s00223-024-01219-y PMC11153283

[20] von Elm E, Altman DG, Egger M, et al The strengthening the reporting of observational studies in epidemiology (STROBE) statement: guidelines for reporting observational studies. Lancet. 2007;370(9596):1453‐1457. 18064739 10.1016/S0140-6736(07)61602-X

[21] Thorsteinsson D, Granath F, Bränström R, Koman A, Zedenius J, Nilsson I-L. Regional variations in the management of primary hyperparathyroidism in Sweden: population-based case-control study. BJS Open. 2024;8(1):zrad154. 38323883 10.1093/bjsopen/zrad154 PMC10848304

[22] Statistics-Sweden . 2017. Background Facts, Population and Welfare Statistics 2017:2, Multi-generation register 2016. A description of contents and quality. Report. ISSN 1654-4331 (Online). Accessed September 10, 2024. URN:NBN:SE:SCB-2017-BE96BR1702ENG_pdf

[23] Ekbom A . The Swedish multi-generation register. Methods Mol Biol. 2011;675:215‐220. 20949391 10.1007/978-1-59745-423-0_10

[24] Thorsteinsson D, Granath F, Branstrom R, Koman A, Zedenius J, Nilsson IL. 2025. Supplementary materials for: Familial aggregation of primary hyperparathyroidism and malignancy—nationwide case-control and cohort study. Zenodo. doi:10.5281/zenodo.15207336 PMC12507010 41069550

[25] Barlow L, Westergren K, Holmberg L, Talbäck M. The completeness of the Swedish cancer register: a sample survey for year 1998. Acta Oncol. 2009;48(1):27‐33. 18767000 10.1080/02841860802247664

[26] Wickham H, Averick M, Bryan J, et al Welcome to the tidyverse. J Open Source Softw. 2019;4(43):1686.

[27] Therneau T . Version 3.8-3. 2024. A Package for Survival Analysis in R. https://CRAN.R-project.org/package=survival

[28] Viechtbauer W . Conducting meta-analyses in R with the metafor package. J Stat Softw. 2010;36(3):1‐48.

[29] Bollerslev J, Rejnmark L, Zahn A, et al European expert consensus on practical management of specific aspects of parathyroid disorders in adults and in pregnancy: recommendations of the ESE educational program of parathyroid disorders. Eur J Endocrinol. 2022;186(2):R33‐R63. 34863037 10.1530/EJE-21-1044 PMC8789028

[30] Wilhelm SM, Wang TS, Ruan DT, et al The American association of endocrine surgeons guidelines for definitive management of primary hyperparathyroidism. JAMA Surg. 2016;151(10):959‐968. 27532368 10.1001/jamasurg.2016.2310

[31] Nilsson IL, Brattsand G, Lunggren Ö, et al Nationellt vårdprogram för primär hyperparatyreoidism. Swedish national guidelines. 2023;(1):10‐12. https://vardpersonal.1177.se/kunskapsstod/

[32] Jha S, Simonds WF. Molecular and clinical Spectrum of primary hyperparathyroidism. Endocr Rev. 2023;44(5):779‐818. 36961765 10.1210/endrev/bnad009 PMC10502601

[33] Cotant CL, Rao PS. Elevated fibroblast growth factor 23 in a patient with metastatic prostate cancer and hypophosphatemia. Am J Kidney Dis. 2007;50(6):1033‐1036. 18037105 10.1053/j.ajkd.2007.07.031

[34] Vargas-Ortega G, Balcazar-Hernandez L, Gonzalez-Virla B, et al Symptomatic primary hyperparathyroidism as a risk factor for differentiated thyroid cancer. J Thyroid Res. 2018;2018:9461079. 30581552 10.1155/2018/9461079 PMC6276394

[35] Li L, Li B, Lv B, et al Increased thyroid malignancy in patients with primary hyperparathyroidism. Endocr Connect. 2021;10(8):885‐893. 34261038 10.1530/EC-21-0217 PMC8346191

[36] Cinamon U, Levy D, Marom T. Is primary hyperparathyroidism a risk factor for papillary thyroid cancer? An exemplar study and literature review. Int Arch Otorhinolaryngol. 2015;19(1):42‐45. 25992150 10.1055/s-0034-1396520 PMC4392524

[37] Norenstedt S, Granath F, Ekbom A, et al Breast cancer associated with primary hyperparathyroidism: a nested case control study. Clin Epidemiol. 2011;3:103‐106. 21487450 10.2147/CLEP.S17298 PMC3072153

[38] Aronoff L, Malkin D, van Engelen K, et al Evidence for genetic anticipation in vonHippel-Lindau syndrome. J Med Genet. 2018;55(6):395‐402. 29437867 10.1136/jmedgenet-2017-104882

[39] von Salomé J, Boonstra PS, Karimi M, et al Genetic anticipation in Swedish Lynch syndrome families. PLoS Genet. 2017;13(10):e1007012. 29088233 10.1371/journal.pgen.1007012 PMC5681299

[40] van den Broek MFM, van Nesselrooij BPM, Pieterman CRC, et al Clues for genetic anticipation in multiple endocrine neoplasia type 1. J Clin Endocrinol Metab. 2020;105(7):dgaa257. 32396602 10.1210/clinem/dgaa257

